# Cancer treatment in Africa: the importance of the role of nursing

**DOI:** 10.3332/ecancer.2019.944

**Published:** 2019-07-25

**Authors:** Anastasia Mitema, Lize Maree, Annie Young

**Affiliations:** 1 Ocean Road Cancer Institute, Ocean Road, Dar es Salaam, Tanzania; 2 Department of Nursing Education, University of the Witwatersrand, Johannesburg 2193, South Africa; 3 Warwick Medical School, University of Warwick, Coventry CV4 7AL, UK

**Keywords:** nursing, leadership, education, cancer treatment pathway

## Abstract

Nurses in all aspects of oncology have a key role to play in the delivery of effective cancer care. Unfortunately, oncology nursing is not yet an established nursing subspecialty in most of Africa; six out of 22 African countries reported as having no trained oncology nurses at all. The need for more personnel and quality training programmes are an absolute necessity.

## Background

Nurses in every country, including those in Africa, form the majority of the healthcare workforce. As a part of the multidisciplinary team, nurses take a leading role in cancer-control programmes locally and country-wide; they are often the first point of contact with their communities in Africa.

Nurses in low- and middle-income countries (LMICs) have a ‘blueprint’ to follow to strive to address the challenges in cancer control [1]. This paper, authored by nurses, outlines the potential impact that oncology-trained nurses can and could make, in finding solutions for service and care improvement in LMICs with partners. In addition, one model to follow and if possible, integrate into practice, is that of the WHO HIV Strategy to End AIDS [2]. Nurses and midwives again are central players in saving many lives [3], through leading innovative and culturally-sensitive practices. The implementation of the recent National Comprehensive Cancer Network Harmonised GuidelinesTM for sub-Saharan Africa [4] provide a huge opportunity for nursing leadership, in particular the guidelines for pain and palliative care [5]. Oncology and palliative care nurses are already showing great leadership skills in tackling the treatment pathway to improve patient care in Africa [6, 7].

Oncology nursing is not yet an established nursing subspecialty in most countries; indeed six out of 22 African countries reported having no trained oncology nurses at all [8]. The social and economic setting, and the political will of African countries are integral to the success of any oncology service [8]. In oncology and palliative care, there are many nurse leaders—‘the’ movers and shakers of policy for Africa. Nurses can become involved in lobbying for change through the National Cancer Associations, the Ministers of Health and the World Health Organisation. Nurse leaders in and for Africa are doing just that—to name but a few: Julia Downing, Professor in Palliative Care, Makerere University, Kampala and Chief Executive, International Children’s Palliative Care Network—for her unstinting work on palliative care, leadership and the evaluation of nurse prescribing of morphine in the palliative care setting [7]; Stella Bialous, immediate past chair of the International Society of Nurses in Cancer Care (ISNCC)—for her tireless campaigning and global nursing interventions on tobacco control in Africa [9]; David Makumi, chair of Kenya Network of Cancer Organisations and Board Member for NCI, Kenya—for his great work in increasing access to breast cancer screening [10]; Lize Maree, Head of Nursing Education, University of Witswatersrand, Johannesburg—a trailblazer in research and education in cancer nursing [11].

The challenges in the delivery of effective cancer treatment in Africa are well documented [8, 12] and have been outlined in previous chapters; unfortunately but perhaps unsurprisingly, ‘poor nursing care’ was identified as a factor in a recent review contributing to a lower survival rate in breast cancer patients [13]. However, the greatest challenges for nurses are similar to the other members of the multidisciplinary team—inadequate treatment facilities and insufficient staff, in particular nurses trained in oncology nursing. Nurses are also low paid and often seek employment in better resourced countries for a higher wage. Human resources development in all fields of cancer control is needed; in Africa, the importance of oncology nursing is not yet recognised. This will change when cancer control in the continent is seen as a problem and put on the health agenda of all African countries.

### Oncology nursing education

Nursing education is the golden key in sub-Saharan Africa [14] but unfortunately, oncology nursing specialisation is not well developed or available. We know that lack of specialised oncology education is the major barrier to effective patient care [15]. Nevertheless, some African countries, such as South Africa and Kenya, have oncology nursing programmes at diploma and masters’ levels. Tanzania, Zambia and Egypt have also made significant progress in developing cancer nursing education [10]. Palliative care education is better established in Africa [16]. Organisations such as ISNCC and the African Organisation for Research and Training in Cancer Nursing Group, through their respective nurse leaders, Patsy Yates and Naomi Oyoe Ohene Oti, are active in developing continent-wide oncology nursing competencies. Informal courses, often supported by oncology nurses from hospitals or universities in high resource countries are abundant but the sustainability of such programmes is questionable.

## Along the cancer treatment pathway

### Surgery

Although nurses are typically not licensed to undertake surgical procedures in African countries, context specific adaptations may allow them to provide front-line surgical care for relatively low-risk surgery (e.g., biopsy of lower female genital tract lesions, including loop electrosurgical excision of the cervix, biopsy of the vulva, endometrial biopsy ultrasound-guided biopsy of palpable breast masses) after adequate training and under supervision [8].

Nurses are the coordinators of care of the patient before, during and after surgery and as a part of the surgical team, monitor the health status of the patient and have the ability to manage acute and chronic surgical-related complications. Post-operative mortality is directly related to the level of nursing care following surgery [17, 18]; this is a good justification for investing in nurse training. Nurses can improve surgical patient care by documenting their patient assessment on instruments developed for the specific purpose as well as applying evidence-based practice. Nurses can also engage in research to develop evidence for their context-specific practice.

### Radiotherapy

Where radiotherapy is available, nurses participate dynamically as a part of the multidisciplinary team in the coordination, education and support of patients undergoing radiotherapy and their families. Most patients develop varying degrees of side effects as a result of radiotherapy during treatment and following completion of treatment. The supportive care required when patients develop expected side effects can be provided by nurses, alongside radiographers. When supportive care is available, this makes a huge difference in the quality of life of cancer patients having radiotherapy. Nurses can improve patient care by documenting their patient assessment on instruments developed for the specific purpose as well as applying and participating in evidence-based practice.

### Chemotherapy

Cost to the patient, lack of cytotoxic drugs on the WHO essential list, unsafe handling and a dearth of experts including nurses to insert central venous catheters, remain the day-to-day barriers to safe administration and handling of chemotherapy for nurses. There are some nurse-led programmes to strengthen the safety aspects of administering chemotherapy in sub-Saharan Africa, e.g., ChemoSafe [19]; we await the evaluation of this programme. Well-trained nurses will improve the quality of cancer care in Africa. Since cancer nurses throughout Africa are frontline care givers, first and foremost communicating with the patients and their families/parents on vital issues, such as striving to complete chemotherapy treatment for their children, is vital. Employing high-quality technical skills in, for example, the administration of cytotoxic drugs, also enhances the quality of patient care. The nurse’s responsibility is great: leading on patient assessment, education, symptom management, and supportive care in addition to administering chemotherapy agents and being responsible for safe handling of drugs, evaluation of laboratory data, calculation of drug dosages, assessing central venous devices, monitoring of adverse reactions interactions and also participating in supportive care research.

## Conclusion

Throughout the cancer treatment pathway in Africa, nurses in Africa have risen to the huge challenge of delivering the best possible care to their patients with few resources. It is heartening to see African nurse leaders emerging to respond to these challenges at every level.

## Conflict of interest

All authors have no conflicts of interest—financial or non-financial—for this article.

## Funding

Anastasia Mitema and Lize Maree have no funding to declare. Funding received by Annie Young in the last 5 years, none of which was related to this article, is as follows:

Honoraria from:

- Bayer
- Leo Pharma
- BMS/Pfizer Alliance

Educational grant from:

- Bayer
